# Plasma Retinol Concentrations and Dietary Intakes of Mother–Infant Sets in Singleton versus Twin Pregnancy

**DOI:** 10.3390/nu15112553

**Published:** 2023-05-30

**Authors:** Anum Akbar, Sarah Duvall, Matthew VanOrmer, Rebecca Slotkowski, Taija Hahka, Thiago Genaro-Mattos, Zeljka Korade, Corrine Hanson, Ann Anderson Berry, Melissa Thoene

**Affiliations:** 1 Department of Pediatrics, University of Nebraska Medical Center, Omaha, NE 68198, USA; anum.akbar@unmc.edu (A.A.); rebecca.slotkowski@unmc.edu (R.S.);; 2 College of Allied Health Professions, University of Nebraska Medical Center, Omaha, NE 68198, USA; 3 Munroe Meyer Institute, University of Nebraska Medical Center, Omaha, NE 68198, USA

**Keywords:** pregnancy, twins, retinol, RAE, vitamin A, maternal, infant, newborn

## Abstract

Vitamin A (retinol) is essential for normal fetal development, but the recommendation for maternal dietary intake (Retinol Activity Equivalent, RAE) does not differ for singleton vs. twin pregnancy, despite the limited evaluation of retinol status. Therefore, this study aimed to evaluate plasma retinol concentrations and deficiency status in mother–infant sets from singleton vs. twin pregnancies as well as maternal RAE intake. A total of 21 mother–infant sets were included (14 singleton, 7 twin). The HPLC and LC-MS/HS evaluated the plasma retinol concentration, and data were analyzed using the Mann–Whitney U test. Plasma retinol was significantly lower in twin vs. singleton pregnancies in both maternal (192.2 vs. 312.1 vs. mcg/L, *p* = 0.002) and umbilical cord (UC) samples (102.5 vs. 154.4 vs. mcg/L, *p* = 0.002). The prevalence of serum-defined vitamin A deficiency (VAD) <200.6 mcg/L was higher in twins vs. singletons for both maternal (57% vs. 7%, *p* = 0.031) and UC samples (100% vs. 0%, *p* < 0.001), despite a similar RAE intake (2178 vs. 1862 mcg/day, *p* = 0.603). Twin pregnancies demonstrated a higher likelihood of vitamin A deficiency in mothers, with an odds ratio of 17.3 (95% CI: 1.4 to 216.6). This study suggests twin pregnancy may be associated with VAD deficiency. Further research is needed to determine optimal maternal dietary recommendations during twin gestation.

## 1. Introduction

Vitamin A (retinol) is a fat-soluble vitamin produced in the body from preformed vitamin A and provitamin A carotenoids found in food [1]. Preformed vitamin A, mainly in the form of retinyl palmitate, is exclusively derived from animal sources [2]. Foods such as milk, meat, and eggs are primary sources of this type of vitamin A. On the other hand, provitamin A carotenoids such α-carotene, β-carotene, and β-cryptoxanthin can be found in both plant-based foods and animal products [3]. The body converts pro-vitamin A carotenoids into retinol. Among these carotenoids, β-carotene has full biological activity, while α-carotene and β-cryptoxanthin exhibit partial biological activity, compared to retinol [4]. Vitamin A in the form of retinoic acid carries out its functions primarily by interacting with nuclear receptors known as retinoic acid receptors (RAR) and retinoid X receptors (RXR) [5]. Upon binding to these receptors, a complex forms between the retinoic acid and its receptors, which subsequently activates the retinoic acid response elements (RAREs) located in the promoter region of target genes. This mechanism allows vitamin A to regulate gene expression, influencing various biological processes.

Vitamin A is an essential micronutrient that plays a vital role in early embryo development, fetal lung development and maturation, cellular differentiation, and fetal organ formation during pregnancy [6,7]. Studies have shown that vitamin A plays a role in regulating cell differentiation and proliferation through epigenetic modifications of DNA methylation and histone modification [8]. However, vitamin A deficiency (VAD) remains one of the leading nutritional deficiencies worldwide, especially among pregnant women [9]. VAD during pregnancy can have various adverse consequences for both mothers and infants [10,11,12,13]. Studies have found an association between VAD and two adverse outcomes in mothers: maternal anemia [11] and night blindness [10]. One possible explanation for the anemia is that vitamin A plays a role in the absorption and utilization of non-heme iron, and a deficiency in vitamin A can lead to anemia [11]. Furthermore, VAD increases susceptibility to infections, and maternal infections may be a contributing factor to preterm delivery in individuals with VAD [12]. In infants, VAD can result in intrauterine growth restriction (IUGR), preterm birth, and increased risk of sepsis [9,12,13].

Maternal metabolic demand during pregnancy is heightened, due to fetal growth. In the case of twin pregnancy, this metabolic demand is further amplified, potentially leading to alterations in nutrient requirements for optimal pregnancy outcomes and a greater probability of encountering nutritional deficiencies in comparison to singleton pregnancy [14]. Worldwide, approximately 2% of all gestations are twin pregnancies, and rates are continuing to rise, due to the growing use of assisted reproductive techniques [15,16]. Twin pregnancy confers several risks to infants and mothers [17,18]. Infants are more prone to preterm birth, leading to potential developmental challenges and health issues. Growth alterations are also common, with an increased likelihood of low birth weight or IUGR [19]. Mothers of twins face a higher susceptibility to gestational diabetes and preeclampsia, posing risks to both mother and babies [17,18]. Additionally, there is a greater chance of maternal or infant morbidity and mortality, due to several factors including the intricate nature of managing multiple pregnancies [15,17]. However, to our knowledge, no study has evaluated the status of retinol in singleton and twin pregnancy and no specific recommendations have been made for vitamin A dietary intake requirements in multiparous pregnancies. Studies comparing the status of vitamin D, folic acid, and energy requirements in singleton vs. twin pregnancy show that the demands for folic acid and vitamin D are higher in twin pregnancies [20]. Notably, however, current maternal dietary recommendations for vitamin A do not differ between singleton and multiparous pregnancies, implying that vitamin A requirements are not increased during twin pregnancy [20,21]. Fetal retinol status is impacted by maternal retinol status and dietary intake [10]; however, there is a gap in our knowledge of how pregnancy type (singleton vs. twin) affects infant retinol status. Therefore, this study aims to analyze maternal RAE intake and retinol concentrations in the umbilical cord (UC) and maternal blood plasma of mother–infant sets from both singleton and twin pregnancies.

## 2. Materials and Methods

### 2.1. Study Design and Participants

After IRB approval, this study performed a secondary analysis of mothers and their infants previously enrolled in a cohort of 687 maternal-infant dyads. Inclusion criteria for the overall cohort included having at least one live-born infant, maternal age of 19 years or older, and admission to the Labor and Delivery Unit at Nebraska Medicine (Omaha, NE, USA) between June 2015 and September 2020. Exclusion criteria included gastrointestinal, liver, or kidney disease affecting nutrient metabolism in mother or infant, inborn errors of metabolism, congenital abnormalities, or infants deemed wards of the state.

After identifying seven twin pregnancies among this initial cohort, we conducted a 2:1 matching process to pair newborns from singleton pregnancies with those from twin pregnancies, resulting in a total sample size of 21 mothers and 28 infants in this subcohort. In the matching process, participants were primarily grouped according to gestational age. In the case of multiple births of the same age, matching was completed stepwise by birth weight, followed by sex, and lastly, race/ethnicity. It should be noted that not all pairs were a perfect match, and some variables were not identical between paired infants.

### 2.2. Biospecimen Collection

Maternal blood plasma and infant umbilical cord plasma samples were collected in K2 EDTA tubes. The maternal blood samples were obtained during regular laboratory procedures when the mother was admitted for delivery. UC blood samples were collected from every newborn during routine cord blood collection at time of delivery. To maintain the nutrient integrity, all blood samples were kept protected from light and heat, and processed and frozen within 12 h of collection at a temperature of −80 °C.

### 2.3. Retinol Laboratory Analysis

A subset (*n* = 22) of the biological samples was analyzed at the Nutritional Biomarker Lab at Harvard T. H. Chan School of Public Health using high-performance liquid chromatography (HPLC) [22]. The other subset (*n* = 6) of samples was analyzed at the University of Nebraska Medical Center using liquid chromatography–tandem mass spectrometry (LC-MS/MS). Plasma retinol concentrations were measured in 100 μL aliquots. The antioxidants and internal standards were added, followed by Folch extraction, separation of the organic phase, and reconstitution in ethanol and acetonitrile. Samples were then placed in an Acquity ultra-performance liquid chromatography (UPLC) system with an ANSI-compliant well plate holder coupled to a Thermo Scientific TSQ Quantis mass spectrometer equipped with an atmospheric pressure chemical ionization (APCI) source. Ten microliters of the sample was injected onto the Phenomenex Luna Omega C18 column using water (0.1% *v*/*v* acetic acid) as solvent A and methanol (0.1% *v*/*v* acetic acid) as solvent B. The total run time was 15 min, at a flow rate of 500 μL/min. The retinol was analyzed using selective reaction monitoring (SRM). Quantitation was achieved using a cocktail of internal standards, and the concentrations were normalized to the amount of sample and reported as mcg/L. Quality control in both analyzing labs was ensured by using NIST standards, and each batch of samples run included several replicates of a plasma pool sample set.

### 2.4. Retinol Status Classification

Maternal plasma retinol concentrations were categorized based on the guidelines provided by the World Health Organization (WHO) into adequate (>300.9 mcg/L), insufficient (≥200.6–300.9 mcg/L), or deficient (<200.6 mcg/L) [23]. For infant UC plasma, there is no clear consensus on the cut-off concentrations for VAD. Thus, in this study, the values used to categorize retinol status in the infant were based on the cut-off points identified by the WHO for extreme cases, which is consistent with other research [24]. Infant UC plasma retinol concentrations were therefore defined as similar to maternal classifications [25].

### 2.5. Electronic Data Collection

The electronic medical record was used to gather data for all mothers and infants in the identified subcohort. From the mother’s medical record, we collected maternal race/ethnicity, delivery mode, pregnancy type (singleton vs. twin), smoking status (current vs. former/never smoker), and mass index (BMI) in kilograms/meters^2^ (kg/m^2^) measured at <10 weeks of gestation. From the infant medical record, we collected infant sex, corrected gestational age at birth (CGA), birth length (centimeters, cm), birth weight (kg) and birth head circumference (cm). CGA groups were defined as preterm (<37 weeks CGA) and term (≥37 weeks CGA), and maternal pre-pregnancy BMI categories were defined using CDC guidelines [26,27].

### 2.6. Maternal Dietary Intake and Recommended Daily Allowance

Maternal dietary intake during pregnancy was evaluated using the Harvard Food Frequency Questionnaire (FFQ), which was completed by the mothers during their hospital stay at the time of delivery [28]. The questionnaire was used to calculate average daily vitamin A intake as measured by retinol activity equivalents (RAE, mcg/day), a standard measure for vitamin A that accounts for pre-formed retinol and provitamin A carotenoid dietary sources [29].

### 2.7. Statistical Analysis

Non-parametric statistical tests were employed for the analysis of continuous and categorical data, due to the small sample size and non-normal data distribution, as confirmed by the Shapiro–Wilk test (*p* < 0.05). The Mann–Whitney U test was used to compare continuous data within and between categorical groups, and Spearman’s rank correlation coefficients were used to evaluate the relationship between continuous variables within and between groups with significant differences. Fisher’s exact and Pearson’s chi-square tests were utilized to determine the association between categorical data. Linear regression analyses were employed to further assess the relationship between variables. The statistical analyses were conducted using IBM SPSS Statistics software (Version 28.0) and a *p*-value < 0.05 was considered significant.

## 3. Results

### 3.1. Study Population

Maternal and infant characteristics are summarized in Table 1 and Table 2, respectively. The results showed no significant differences between singleton- and twin-pregnancy types, in terms of maternal age at delivery, BMI, race/ethnicity, mode of delivery, or smoking habits (*p* > 0.05). Additionally, there were no significant differences in infant characteristics, including birth CGA, birthweight, birth length, head circumference, or sex, between singleton- and twin-pregnancy types (*p* > 0.05). All mothers from both groups met the recommended intake of 770 mcg/d of RAE, with one mother in the singleton-pregnancy type exceeding the maximum dose or upper limit (UL) of 3000 mcg/d, by consuming 3206 mcg/d (107% of UL) [29]. Moreover, there was no significant difference found in maternal RAE intake between the groups (*p* = 0.603), with a median RAE intake of 1862 mcg/day and 2178 mcg/day in mothers of singletons vs. mothers of twins, respectively.

### 3.2. Retinol Status in Pregnancy Types

Table 3 displays the plasma retinol concentrations for the maternal and UC samples, as categorized by pregnancy type. Mothers of twins had significantly lower plasma retinol concentrations than mothers of singletons (192.2 mcg/L vs. 312 mcg/L, *p* = 0.012). Likewise, infant UC plasma retinol concentrations were significantly lower in twins compared to singletons (103 mcg/L vs. 154 mcg/L, *p* = 0.002). However, no significant difference was found between infant UC plasma retinol concentrations for twin 1 and twin 2 (106.9 mcg/L vs 100.9 mcg/, *p* = 0.383) (not shown in Table 3) and most of the mothers in our study cohort had pregnancies with diamniotic and dichorionic twins.

### 3.3. Vitamin A Status between Pregnancy Types

Table 4 presents the proportions of adequate, insufficient, and deficient categorizations of plasma retinol for both mothers and infants. The results demonstrate significant differences in the proportion of classifications by pregnancy type for both mothers (*p* = 0.031) and infants (*p* < 0.001). In mothers, only 7% of singletons were VAD, compared to 57% of twin pregnancies (*p* = 0.025). However, there were no significant differences between classifications of adequacy (*p* = 1.00) or insufficiency (*p* = 0.159).

Among infants, all 14 twin newborns but no singleton newborns met the criteria for VAD (*p* < 0.001). As such, there was a significant difference in the number of singleton newborns meeting the criteria for insufficiency, compared to twin newborns (*p* < 0.001).

### 3.4. Relationship between Maternal Retinol Concentrations and Pregnancy Type on Infant Retinol Concentrations

A significant positive correlation was found between maternal plasma and infant UC plasma retinol concentrations in singleton pregnancies (r = 0.574, *p* = 0.032). However, there was no significant correlation found between the same variables in twin pregnancies (r = 0.408, *p* = 0.148). No significant correlations were identified between maternal plasma retinol and maternal RAE intake in singleton or twin pregnancies (r = 0.288, *p* = 0.318 and r = 0.071, *p* = 0.808, respectively) or between infant UC plasma retinol and maternal RAE intake in singleton or twin pregnancies (r = −0.018, *p* = 0.714 and r = −0.266, *p* = 0.358, respectively) (Table 5). Spearman’s rank correlation coefficients showed a lack of a significant relationship between the UC plasma retinol concentration of twin 1 and twin 2 (r = 0.643 and *p* = 0.119) in a singular set. Similarly, correlations between birth CGA and infant UC plasma retinol or maternal plasma retinol were both found to be insignificant (*p* = 0.895 and *p* = 0.130).

The results of linear regression analysis indicate that singleton pregnancy is associated with significantly higher UC plasma retinol levels, with a beta value of 63.73 (*p* < 0.05). This suggests that, on average, singleton pregnancy UC plasma retinol is 63.73 higher when compared to twin pregnancy. The pregnancy-type variable was found to account for 21.7% of the variance in the UC plasma retinol levels (r^2^ = 0.217, *p* < 0.05), indicating that pregnancy type is a significant predictor of retinol levels. Moreover, the odds of vitamin A deficiency in mothers with twin pregnancies were found to be significantly higher compared to singleton pregnancies, with a 17.3-fold increase in odds (95% confidence interval: 1.4–216.6). This suggests that twin pregnancy is associated with a substantially greater risk of VAD in mothers.

## 4. Discussion

### 4.1. Retinol Status

Twin pregnancy was identified as a possible risk factor for VAD in infants. To our knowledge, this is the first study to report this finding. No singleton infants had VAD, which conflicts with other studies. We have previously reported high rates of VAD in singleton infants [30]. However, this study observed striking VAD among the twin participants in this cohort. While there is limited information on plasma retinol concentrations in twin pregnancies, similar results have been found in the case of other micronutrients [31]. A prior study found that twin newborns and their mothers had higher rates of vitamin D deficiency compared to singletons. The transfer of nutrients through the placenta is believed to contribute to VAD, particularly in the context of twin pregnancies, where there may be two placentas [32]. This is because each placenta needs to fulfill the nutritional needs of one fetus with a singular maternal nutrient supply, and each placenta also retains retinol within its tissue, combining to result in low plasma retinol levels in twin infants. In this study, it was found that 6 out of 7 twin sets had diamniotic dichorionic pregnancies (the most common type of twin placentation), which could have also contributed to the observed low concentrations of plasma retinol in infants.

Similarly, mothers of twins had a higher prevalence of VAD than singleton mothers, which is theorized to be due to increased nutrient requirements during twin compared to singleton pregnancy, depleting retinol stores more quickly by the time of delivery [33]. It is possible that having to nourish two babies instead of one contributes to this difference. In addition, the difference in plasma retinol concentration between twin and singleton pregnancies may also be due to variations in the concentration of the retinol binding protein (RBP4), which has been shown in previous research to affect retinol concentrations in singleton pregnancies [34]. Not surprisingly, twin newborns did not have significantly different plasma retinol concentrations within individual twin sets (twin 1 vs. twin 2).

Even though RAE intake was within recommended limits in both groups, twin mothers and infants were still found to be deficient in vitamin A. In addition, the study found that there was no significant difference in maternal RAE intake between mothers of singletons and twin pregnancies, and no significant correlations were identified between maternal and infant UC plasma retinol and maternal RAE intake in singleton or twin pregnancies. These findings suggest that there may be differences in the relationship between maternal and infant retinol concentrations in singleton versus twin pregnancies, and that maternal RAE intake may not be the only contributor to infant UC plasma retinol concentrations. In addition, it should be noted that the status of maternal retinol does not always have an impact on infant retinol stores, as there are several factors that can affect this status [35]. Therefore, increasing dietary retinol intake may not necessarily result in an immediate increase in maternal plasma or infant umbilical cord plasma retinol concentrations, since the metabolism and distribution of the nutrient can vary throughout the body [36]. Nonetheless, it is crucial for pregnant women to meet the recommended intake of retinol to fulfill their physiological needs, decrease the risk of VAD, and provide sufficient supply to their newborn infants during breastfeeding [6].

It is also important to note that excessive exposure to vitamin A could lead to vitamin A toxicity, although such instances are rare in the US. Vitamin A toxicity can be categorized into two main forms: acute and chronic [37]. Acute vitamin A toxicity predominantly affects the mucocutaneous system, while chronic vitamin A toxicity can impact multiple organ systems. Of particular concern during fetal development is the teratogenicity caused by excessive vitamin A, which can lead to various congenital anomalies affecting multiple systems, including the central nervous system (CNS), cardiovascular system (CVS), and genitourinary system [38]. Conversely, mothers experiencing acute vitamin A toxicity may exhibit symptoms such as nausea, vomiting, increased cerebrospinal fluid pressure, headaches, blurred vision, and impaired muscular coordination [39]. Consequently, interventions aimed at increasing maternal RAE intake should consider focusing on provitamin A carotenoids as a safer alternative to preformed vitamin A [38].

Unlike preformed vitamin A, provitamin A carotenoids are generally considered safe, even at high intake levels. A possible reason for this disparity is a difference in the intestinal absorption of preformed vitamin A via passive diffusion, whereas provitamin A carotenoids are absorbed via protein-mediated transport [40]. With passive diffusion, a higher intake of vitamin A will lead to more absorption of vitamin A, hence increasing the chances of vitamin A toxicity. However, with protein-mediated transport, absorption is limited once the transport proteins are saturated, preventing high intakes of provitamin A carotenoids from causing vitamin A toxicity. This characteristic renders provitamin A carotenoids a potentially beneficial strategy for preventing VAD during pregnancy. By promoting the consumption of provitamin A carotenoid-rich foods, such as fruits and vegetables, interventions can help ensure an adequate vitamin A supply to pregnant women while minimizing the risk of excessive retinol intake. Until more information becomes available, women with multiparous pregnancies should continue to adhere to the current recommendations for dietary RAE intake.

### 4.2. Strengths and Limitations

This study has several notable strengths, including its novel exploration of retinol status among mother–infant sets of singletons vs. twin pregnancies and the use of a matching process to control confounding variables. However, there are also several limitations to consider. The sample size was relatively small, and certain samples were analyzed at different labs—although NIST standards were used in each. Additionally, factors such as socioeconomic status and smoking were not adjusted for in the analysis, despite potentially affecting retinol levels. Furthermore, the matching process based on CGA may have skewed the singleton group towards premature infants—factors such as group B streptococcus infection, advanced maternal age, and a history of alcohol and smoking were found to be associated with preterm delivery in singleton pregnancies—which could be influenced by the shorter gestational period of twin pregnancies. It is important to note that retinol status assessment is challenging in infants, due to low sample volumes, and the WHO criteria for VAD is targeted towards older populations [23,41]. Therefore, some limitations need to be considered when interpreting the study’s findings.

## 5. Conclusions

This study has revealed that the plasma retinol concentrations of mother–infant sets at the time of delivery are impacted by multiparity, as twin mothers and infants demonstrated a higher prevalence of VAD. Although the study did not establish a link between maternal vitamin A consumption and plasma retinol concentrations, it highlights the need for additional research to identify age-specific plasma retinol values for infants, which may indicate health risks associated with VAD. Future studies could also evaluate whether dietary RAE intake recommendations for singleton vs. multiparous pregnancies differ, and analyze larger populations of term versus preterm individuals in singleton versus twin pregnancies.

## Figures and Tables

**Table 1:** Baseline Characteristics of Mothers by Pregnancy Type.

Maternal Characteristics (*n* = 21)	Singleton Mothers (*n* = 14)		Twin Mothers (*n* = 7)		*p*-Value
	Median	IQR	Median	IQR	
Age at delivery (years)	31	26.25–36.25	32	28.0–36.0	0.946
BMI ** (kg/m^2^)	30.9	24.7–34.6	31.2	26.0–38.9	0.202
Maternal RAE intake *** (mcg/day)	1862	1256.5–2371.0	2178	1606.3–2342.8	0.603
	*n* (%)		*n* (%)		
Race					
White	9 (64%)		5 (57%)		1.00
Non-white	5 (36%)		3 (43%)		
Mode of Delivery					
Vaginal	6 (43%)		3 (43%)		1.00
Cesarean	8 (57%)		4 (57%)		
Smoking Status					
Current smoker	3 (21%)		1 (14%)		1.00
Former/Never smoker	11 (79%)		6 (86%)		

** For singleton pregnancies, pre-conception BMI was available for 8 mothers. For twin pregnancies, pre-conception BMI was available for 6 mothers. *** RAE: Retinol Activity Equivalent.

**Table 2:** Baseline Characteristics of Singleton and Twin Newborns.

Infant Characteristics (*n* = 28)	Singleton Newborns (*n* = 14)		Twin Newborns (*n* = 14) **		*p*-Value
	Median	IQR	Median	IQR	
Corrected Gestational Age (CGA) (weeks)	36.6	32.7–37.6	36.6	32.9–37.6	0.804
Birth weight (kg)	2.4	2.2–3.0	2.4	1.8–3.2	0.874
Birth length (cm)	45.3	44.4–48.5	46.8	43.4–48.3	0.804
Head circumference (cm)	32.9	31.9–34.5	32.8	30.8–34.5	0.804
	*n* (%)		*n* (%)		
Sex					
Male	12 (86%)		8 (57%)		0.209
Female	2 (14%)		6 (43%)		

** Characteristics of twins are noted as individuals, not as sets, so each twin infant’s characteristics are represented.

**Table 3:** Differences in Maternal and Infant Plasma Retinol Concentrations Between Singleton and Twin Pregnancies.

	Singletons (*n* = 14)		Twins (*n* = 14)		*p*-Value
	Median	IQR **	Median	IQR **	
Maternal Plasma Retinol (mcg/L)	312.1	262.5–396.5	192.2	181.2–297.6	0.012
Infant UC *** Plasma Retinol (mcg/L)	154.4	123.1–211.2	102.5	96.4–137.1	0.002

** IQR: Interquartile Range. *** UC: Umbilical Cord.

**Table 4:** Categorizations of Retinol Status of Mothers and Infants Between Pregnancy Type.

	Singleton Pregnancy (*n* = 14)	Twin Pregnancy (*n* = 7)	*p*-Value
Mothers (*n* = 21)	*n* (%)	*n* (%)	0.031
Deficient (<200.55 mcg/L)	1 (7%)	4 (57%)	
Insufficient (≥200.55–300.82 mcg/L)	5 (36%)	2 (29%)	
Adequate (>300.82 mcg/L)	8 (57%)	1 (14%)	
	**Singleton Pregnancy (*n* = 14)**	**Twin Pregnancy (*n* = 14)**	***p*-Value**
Infants (*n* = 28)	*n* (%)	*n* (%)	<0.001
Deficient (<100.27 mcg/L)	0 (0%)	14 (100%)	
Insufficient (≥100.27–300.82 mcg/L)	13 (93%)	0 (0%)	
Adequate (>300.82 mcg/L)	1 (7%)	0 (0%)	

**Table 5:** Correlations of Maternal and Infant Retinol Plasma Concentrations with Other Variables.

Retinol Concentration	Correlations with	Single Pregnancy		Twin Pregnancy	
		Spearman’s R	*p*-Value	Spearman’s R	*p*-Value
Maternal Plasma Retinol (mcg/L)	UC *** Plasma Retinol	0.574	0.032	0.408	0.148
	Maternal RAE **	0.288	0.318	0.071	0.808
UC *** Plasma Retinol (mcg/L)	Maternal RAE **	−0.108	0.714	−0.266	0.358

** RAE: Retinol Activity Equivalents. *** UC: Umbilical Cord.

## Data Availability

Data is available upon request to the corresponding author.
